# Evaluation of Glycosylated PTGS2 in Colorectal Cancer for NSAIDS-Based Adjuvant Therapy

**DOI:** 10.3390/cells9030683

**Published:** 2020-03-11

**Authors:** Roberta Venè, Delfina Costa, Raffaella Augugliaro, Sebastiano Carlone, Stefano Scabini, Gianmaria Casoni Pattacini, Maurizio Boggio, Simonetta Zupo, Federica Grillo, Luca Mastracci, Francesca Pitto, Simona Minghelli, Nicoletta Ferrari, Francesca Tosetti, Emanuele Romairone, Maria C. Mingari, Alessandro Poggi, Roberto Benelli

**Affiliations:** 1 OU Molecular Oncology & Angiogenesis, IRCCS Ospedale Policlinico San Martino, Largo Rosanna Benzi 10, 16132 Genoa, Italy; roberta.vene@hsanmartino.it (R.V.); delfina.costa@hsanmartino.it (D.C.); nicoletta.ferrari@hsanmartino.it (N.F.); francesca.tosetti@hsanmartino.it (F.T.); alessandro.poggi@hsanmartino.it (A.P.); 2 OU Immunology, IRCCS Ospedale Policlinico San Martino, Largo Rosanna Benzi 10, 16132 Genoa, Italy; raffaella.augugliaro@hsanmartino.it (R.A.); mariacristina.mingari@unige.it (M.C.M.); 3 OU Cell Biology, IRCCS Ospedale Policlinico San Martino, Largo Rosanna Benzi 10, 16132 Genoa, Italy; sebastiano.carlone@hsanmartino.it; 4 OU Oncologic Surgery and Implantable Systems, IRCCS Ospedale Policlinico San Martino, Largo Rosanna Benzi 10, 16132 Genoa, Italy; stefanoscabini@libero.it (S.S.); gianmaria.casonipattacini@gmail.com (G.C.P.); 5 OU Pathology, IRCCS Ospedale Policlinico San Martino, Largo Rosanna Benzi 10, 16132 Genoa, Italy; maurizio.boggio@hsanmartino.it (M.B.); federica.grillo@unige.it (F.G.); mastracc@hotmail.com (L.M.); pitto.francesca@yahoo.it (F.P.); 6 OU Molecular Diagnostics, IRCCS Ospedale Policlinico San Martino, Largo Rosanna Benzi 10, 16132 Genoa, Italy; simonetta.zupo@hsanmartino.it; 7 Department of Surgical Science and Integrated Diagnostics, University of Genoa, 16132 Genoa, Italy; 8 Clinical and Experimental Immunology lab, Ospedale G. Gaslini, 16147 Genoa, Italy; simoming@tin.it; 9 Department of General Surgery, Asl3, Ospedale Villa Scassi, 16149 Genoa, Italy; emanuele.romairone@asl3.liguria.it; 10 Department of Experimental Medicine (DIMES), University of Genoa, 16132 Genoa, Italy

**Keywords:** prostaglandin-endoperoxide synthase-2/Cyclooxygenase 2 (PTGS2/COX-2), colorectal cancer (CRC), non-steroidal anti-inflammatory drugs (NSAIDS), cancer-associated fibroblasts (CAF), interleukin-1 beta (IL1β), macrophages

## Abstract

Observational/retrospective studies indicate that prostaglandin-endoperoxide synthase-2 (PTGS2) inhibitors could positively affect colorectal cancer (CRC) patients’ survival after diagnosis. To obtain an acceptable cost/benefit balance, the inclusion of PTGS2 inhibitors in the adjuvant setting needs a selective criterion. We quantified the 72 kDa, CRC-associated, glycosylated form of PTGS2 in 100 frozen CRC specimens and evaluated PTGS2 localization by IHC in the same tumors, scoring tumor epithelial-derived and stroma-derived fractions. We also investigated the involvement of interleukin-1 beta (IL1β) in PTGS2 induction, both in vitro and in CRC lysates. Finally, we used overall survival (OS) as a criterion for patient selection. Glycosylated PTGS2 can be quantified with high sensibility in tissue lysates, but the expression in both tumor and stromal cells limits its use for predictive purposes. Immunohistochemistry (IHC) analysis indicates that stromal PTGS2 expression could exert a protective role on patient OS. Stromal PTGS2 was prevalently expressed by cancer-associated fibroblasts exerting a barrier function near the gut lumen, and it apparently favored the antitumor M1 macrophage population. IL1β was directly linked to gPTGS2 expression both in vitro and in tumors, but its activity was apparently prevalent on the stromal cell population. We suggest that stromal PTGS2 could exert a positive effect on patients OS when expressed in the luminal area of the tumor.

## 1. Introduction

Prostaglandin-endoperoxide synthase-2 (PTGS2), one of the key enzymes mediating prostaglandins neosynthesis, is typically induced by inflammatory stimuli and expressed by tumor epithelial cells in about 74–78% of colorectal cancer (CRC) (see [1] for review). PTGS2 exists both as a rapidly-degraded 68 kDa unglycosylated form, with increased catalytic activity, and a more stable, endoplasmic reticulum-associated, 72 kDa glycosylated form (gPTGS2) [2]. While unglycosylated PTGS2 can be detected in the normal mucosa, gPTGS2 is typically associated with CRC.

PTGS2 has been considered an ideal target for colorectal tumor chemoprevention [3,4], but the cardiotoxicity associated to the specific PTGS2 inhibitor Celecoxib determined an unfavorable cost/benefit ratio for the chemoprevention of the normal population. On the contrary, the inhibition of PTGS2 in the adjuvant setting could be beneficial for CRC patients. Three independent observational studies by Ng, Hua, and Friis indicated an increased survival for long-term, regular users of non-steroidal anti-inflammatory drugs (NSAIDS) after CRC diagnosis [5,6,7], of which specific PTGS2 inhibitors were found to be the most active. At present, Celecoxib is prospectively tested in patients with resected stage III colon cancer and treated with adjuvant FOLFOX chemotherapy (https://clinicaltrials.gov/ct2/show/NCT01150045). This phase III, multicenter trial will give a fundamental hint for the rational use of PTGS2 inhibitors in advanced CRC. Nevertheless, a limit of this study and of future applications is the lack of criteria for patient selection. The influence of tumor PTGS2 expression on CRC patient prognosis is difficult to interpret [8,9,10,11]. Moreover, despite the influence of tumor stroma and leucocyte infiltration in CRC progression [12,13,14], previous studies did not evaluate the influence of PTGS2 expressed by non-tumor cells on patient prognosis. We here quantified the 72 kDa gPTGS2 in 100 primary CRC lysates as a proof of principle for the identification of patients that could benefit from NSAIDS treatment after surgery. PTGS2 levels were also evaluated by the same antibody in immunohistochemistry (IHC), distinguishing tumor-derived from stroma-derived PTGS2. We also evaluated IL1β as a candidate of inflammation-driven stromal PTGS2 expression. PTGS2 was finally correlated with patient prognosis to evaluate its association with CRC aggressiveness.

## 2. Materials and Methods

### 2.1. Patients

The study was conducted in accordance with the Declaration of Helsinki, and the protocol was approved by the Ethics Committee of San Martino Hospital (Ethical code number: n°4/2011). All subjects were recruited at the unit of Oncologic Surgery and Implantable Systems after giving their informed consent. A total of 100 patients subjected to surgical resection of CRC by dedicated surgeons were included (49 males and 51 females; median age 70 years). All patients underwent surgery as the first curative treatment. Tumors were located in the ascending (41), transverse (2), descending (17), sigmoid colon (19), and rectum (21), and they were staged as I (14), II (34), III (39), and IV (13) according to Union for International Cancer Control (UICC) 2009 classification.

### 2.2. Specimens Collection and Processing

Each surgical specimen was collected within 20 min after resection and evaluated by an expert pathologist who collected a representative fragment of the invasive tumor and a strip of normal mucosa, which was sampled at least 10 cm from the tumor mass. Each sample was placed in Safe-Lock tubes (Eppendorf Srl, Milan, Italy) with 80 µL of RIPA buffer containing sodium orthovanadate (OV) 1 mM, dithiothreitol (DTT) 1 mM and a protease inhibitor cocktail 1:100 (Sigma-Aldrich Italia, Milan, Italy, P8340), and stored at −80 °C. Frozen tissues were thawed on ice and minced with sharp scissors, adding 100 µL of fresh RIPA buffer (with OV, DTT and protease inhibitors) to each sample. After 90 min incubation on ice, samples were potterized and centrifuged (24,000× *g*, 4 °C). Supernatants were collected and protein content was quantified by the DC protein assay (Bio-Rad Laboratories Srl, Milan, Italy).

### 2.3. Cell Lines

CaCo2 and HT29 (PTGS2 positive), SW480 and HCT15 (PTGS2 negative), DLD1, SW620, LS180 human CRC cell lines (obtained from the Biological Bank of our institute, http://www.iclc.it), and MF2T colon fibroblast primary cell culture [15] were cultured in RPMI 10% FCS.

### 2.4. Western Blot

Additional information about antibody selection and Western blot quantification of PTGS2 is available in Supplementary method S1.

All 100 samples from normal mucosa and cancer tissue (= 200 samples) were analyzed. Total proteins (30 µg/lane) were resolved on 10% SDS PAGE precast gels (Thermo Scientific) and blotted on PVDF membranes (GE-healthcare Italia, Milan, Italy). Anti PTGS2 (D5H5) rabbit mAb and HRP-conjugated (goat anti-rabbit 7074S) secondary antibody were from Cell Signaling Technology, Leiden, The Netherlands). HRP-conjugated anti beta-actin (13E5 rabbit mAb, Cell Signaling Technology) was used as loading control. Protein bands were detected by a chemiluminescent HRP substrate (Immobilon Western, Merk Life Science Srl, Milan, Italy) and acquired by a C-Digit blot scanner (LI-COR, Bad Homburg, Germany). Only the 72 kDa gPTGS2 band was quantified by the Image Studio 4.0 software. All blots were normalized against two CaCo2 internal standards (10 and 30 µg) loaded in each blot. gPTGS2 relative values were normalized extracting the cubic root (CBRT) of each value to allow the application of parametric statistics. Human PTGS2 standard (cat. 100200-4 Alpha Diagnostic International, San Antonio, TX, USA) was used to estimate gPTGS2 concentration in 30 µg of total tissue lysate.

In vitro studies: in the first test, MF2T primary fibroblasts from human colon were serum-starved for 48 hours and treated with interleukin 8 IL8/CXCL8 (Peprotech, London, UK 10 ng/mL), PGE2 (Cayman, Ann Arbor, MI, USA, 100 nM), growth-regulated oncogene beta GROβ/CXCL2 (Peprotech, 10 ng/mL), interleukin-1 beta (IL1β, Peprotech, 0.1 ng/mL), or epithelial growth factor EGF (Peprotech, 10 ng/mL) for 24 hours to verify the leading role of IL1β in PTGS2 induction (tested in duplicate). In the second test, CRC cell lines were treated with 0.1 ng/mL IL1β in the same conditions of MF2T (tested in duplicate). Cells were scraped, washed once in PBS, and immediately lysed in RIPA buffer. Western blot was run as reported above for tissue samples.

### 2.5. Immunohistochemistry

A pathologist identified the representative paraffin-embedded tumor sample for each of the 100 cases analyzed by western blot (WB). Four µm thick sections were cut and mounted on Superfrost slides (Thermo-Fisher Scientific Italia, Milan, Italy). IHC was carried out with the automated BenchMark Ultra Immunostainer® (Ventana Medical Systems Tucson, Arizona, USA). Primary, anti-PTGS2 (D5H5) rabbit mAb was used at 1:100 final dilution and detected by Ultraview universal DAB detection kit (Ventana Medical Systems).

PTGS2 was scored by a trained pathologist as the percent of positive cells for both stromal and tumor epithelial cells. Necrotic tissue was excluded from evaluation. Percentages were subdivided into three categories (Low, Medium, High) for Kaplan–Meier analysis. Positivity was classified as follows: negative or barely distinguishable staining, or ≤5% = Low; >5% to ≤20% = Medium; >20% = High.

PTGS2 positive cells in tumor stroma were further characterized either as serial sections stained with DAB for bright field microscopy (CD68 and CD163 positive macrophages: 85 hot-spots in 33 samples), or by the double-fluorescent staining of single sections (Vimentin-positive mesenchymal cells, 14 samples). Samples were probed with the following antibodies: anti PTGS2 D5H5 mAb, anti vimentin (1:100, Thermo-Fisher Scientific Italia), anti CD68 (1:500, BioCare, Pacheco, CA, USA), anti CD163 (1:300, BioCare), using a Leica Bond-RX immunostainer. Images were captured by a Leica AT2 scanner (bright field) or Leica DM-LB2 microscope (Leica Biosystems, Milancity, Italy) equipped with a GXCam-U3-18 camera (fluorescence). The percent of PTGS2, CD68, and CD163-positive cells in hot spots was quantified by the Image Scope 12.3 software (Leica). PTGS2-vimentin fluorescent co-localization was analyzed by the JACoP plug-in of ImageJ (https://imagej.nih.gov/ij/plugins/track/jacop2.html).

Multiplexed IHC on single tissue sections was performed using AEC (Enzo life sciences, Farmingdale, NY, USA) as the chromogenic substrate. Anti mannose receptor 1 (MRC1) (Sigma-Aldrich Italia, prestige antibody AMAb90746; 1:5000 dilution), anti inducible nitric oxide synthase (iNOS) (Thermo-Fisher Scientific Italia, PA3-030A; 1:600 dilution) and anti arginase 1 (ARG1) (Sigma-Aldrich Italia, prestige antibody HPA003595; 1:2000 dilution) were tested in addition to anti CD68 and anti CD163. The first multiplex tested CD68–iNOS–PTGS2 consecutively (7 samples–36 fields–108 images), the second multiplex ARG1–MRC1–CD163–PTGS2 (7 samples–44 fields–176 images). Controls of complete destaining were performed using only the secondary antibody between iNOS and PTGS2 in the first series and MRC1 and CD163 in the second series: no signal was detected. After AT2 slide scanning of each single staining, slides were destained by ETOH washings (5 min 50% ETOH, 10 min 100% ETOH, 5 min 50% ETOH) and antibodies were removed by a guanidine-based stripping solution (6 M Gn-HCl, 0.2% NP-40, 10 mM DTT, 20 mM Tris-HCl, pH7.5; 37 °C, 20 min). After extensive washing in running tap water, (5’) slides were probed with the successive primary antibody. Hematoxylin was used for nuclear counterstaining only in the first run. AEC positive staining was identified in each slide using Image-J color thresholds and extracted as 8-bit black&white mask (see Supplementary Figure S1), the colocalization of markers (1:1) was quantified by JACoP plug in.

### 2.6. IL1β ELISA

IL1β was quantified in tumor tissue lysates from 60 unselected cases by RayBio Human IL1 beta ELISA (RayBiotech, Peachtree Corners, GA, USA, ELH-IL1β) according to the manufacturer’s instructions; 15 µg of tissue lysate in 100 µL of diluent was plated in each well (test run in duplicate). 

### 2.7. Statistics

All analyses were performed using the free statistical software EZR 1.41 (http://www.jichi.ac.jp/saitama-sct/SaitamaHP.files/statmed.html). The analysis of variance among three or more groups of data was performed by one-way ANOVA or Kruskal–Wallis test. Correlations were calculated by Pearson’s or Spearman’s test. Nominal data distribution was analyzed by Fisher’s exact test. Patients’ survival was analyzed by Kaplan–Meier analysis and a Long-rank test. A *p* ≤ 0.05 was considered statistically significant.

## 3. Results

### 3.1. gPTGS2 Quantification in 100 CRC Lysates and its Relation to Tissue PTGS2

**Figure 1:**
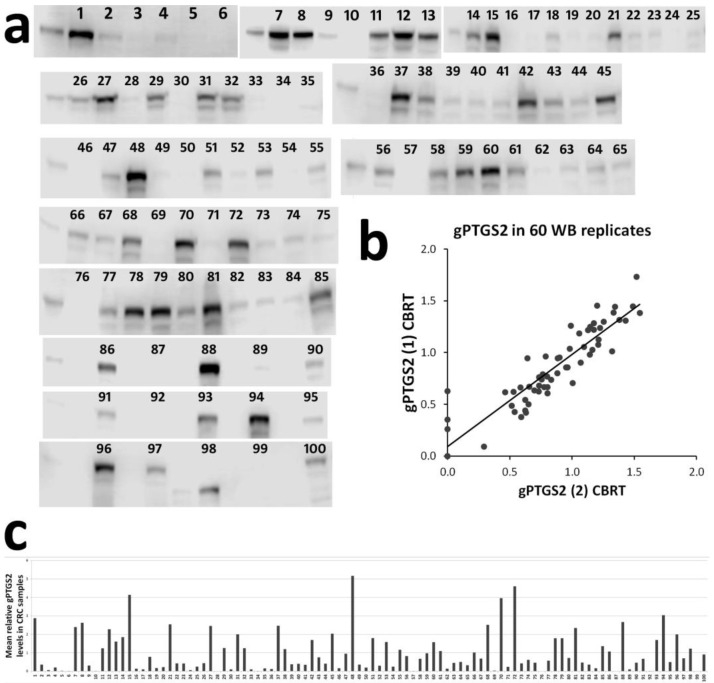
Western blot quantification of glycosylated prostaglandin-endoperoxide synthase-2 (gPTGS2) in colorectal cancer (CRC) lysates: (**a**) c-digit-extracted pseudo-images showing gPTGS2 signal in 100 CRC samples; (**b**) reproducibility of PTGS2 quantification, by replicated WB analysis, in 60 CRC samples; (**c**) relative quantification of gPTGS2 levels in CRC samples.

gPTGS2 was detectable by WB in 96/100 CRC (Figure 1a,c) (median = 156.86 pg, mean = 293.3 pg, range 0.00–1515.64 pg of protein, in 30 µg of tissue lysate, according to the hu PTGS2 standard) and in 11/100 of matched normal mucosa (median = 0.00 pg, mean = 0.003 pg, range 0.00–79.8 pg of protein, in 30 µg of tissue lysate). Compared to other studies (see Supplementary Table S1), this is a high detection rate. The replicate of WB analysis on 60 CRC (Figure 1b) showed a high correlation (Pearson’s correlation r = 0.907, p = 0.0000000000000000000000217, ensuring sufficient reproducibility.

**Figure 2:**
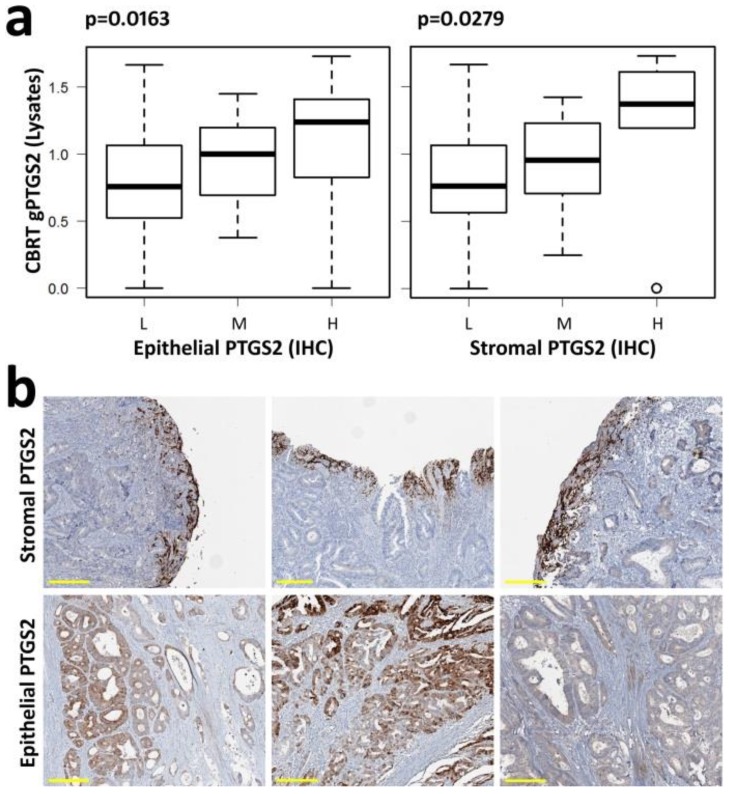
gPTGS2 is expressed in tumor stroma and tumor epithelial cells: (**a**) gPTGS2 levels, quantified in 100 CRC lysates by WB, show a directly proportional correlation with both epithelial and stromal PTGS2 scored by IHC (L = low, M = intermediate, H = high PTGS2 expression); (**b**) PTGS2 positive stromal cells show a bright staining and localize close to the outer mucosal layer of the tumor (upper row), suggesting a protective, barrier function. Tumor epithelial positivity can be observed in all tumor areas, with variable intensity of staining and localization (lower row). Yellow scale bar = 200 µm.

PTGS2 was also evaluated by IHC in 100 matched CRC paraffin embedded tissues, using the same primary antibody. Tumor-associated and stroma-associated PTGS2 were scored independently. The correlation coefficient of tumor PTGS2 compared with stromal PTGS2 was 0.334 (Spearman’s rank, *p* < 0.001). Thus, the contemporary presence of high or low PTGS2 levels in the tumor and stromal populations of the same sample was apparently infrequent in our cohort, suggesting the existence of distinct mechanisms of PTGS2 induction in the different cell populations of the same tumor. In tissue lysates, both tumor and stromal cells contributed to total gPTGS2 levels, showing a directly proportional correspondence with IHC data (Figure 2a).

PTGS2-positive cells of the stromal component almost invariably localized in the luminal area of the tumor, with a strong intensity of staining. These cells frequently lined the limit between living tissue and necrotic areas or surrounded crypts of the outer epithelial border (Figure 2b), suggesting a protective function. In CRCs with medium–high PTGS2 epithelial staining, an irregular distribution of positive areas was observed (Figure 2b). 

### 3.2. Identification of gPTGS2 Positive Cells in the Stromal Component

**Figure 3:**
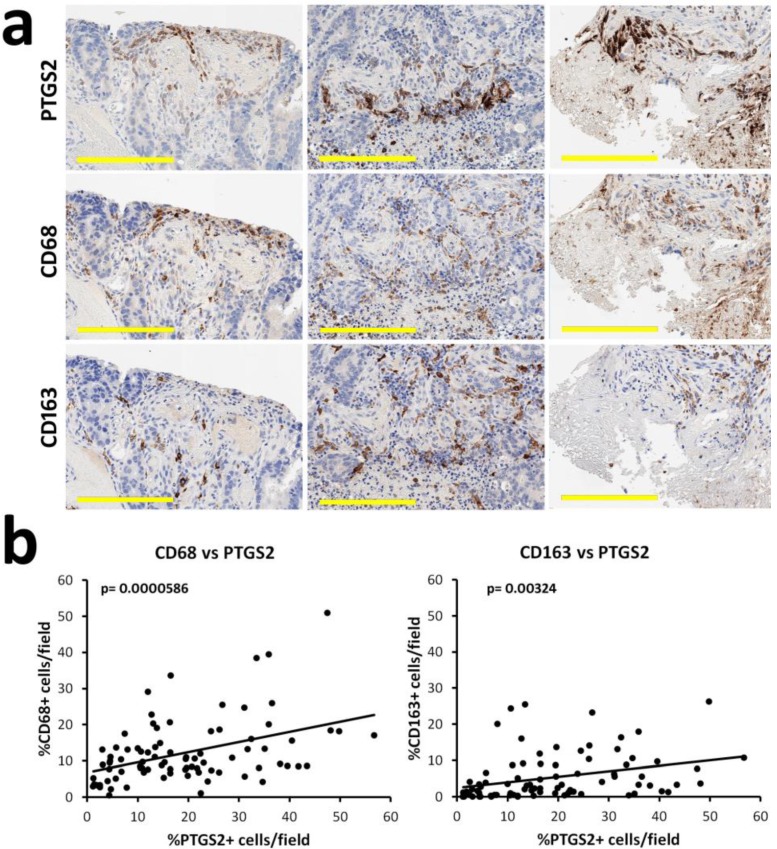
Analysis of PTGS2 influence on CD68 and CD163 macrophage populations on CRC serial sections: (**a**) Representative images of PTGS2, CD68, and CD163 staining on serial sections: PTGS2 can be found in areas enriched with CD68-positive macrophages, but it shows infrequent overlapping. Scale bar = 200 µm; (**b**) Digital pathology quantification of PTGS2, CD68, and CD163-positive cells on overlapping areas of serial sections and plot of linear regression. PTGS2 showed a partial relation with CD68-positive macrophages and a weak relation with the CD163 counterpart.

As in our CRC cohort, PTGS2-positive stromal populations with a luminal distribution were previously observed in colon adenomas: Chapple and Bamba independently attributed PTGS2 positivity to macrophages, according to cell morphology or CD68 expression [16,17]. Tumor-infiltrating macrophages have been classified as M1 (antitumor) or M2 (protumor) according to the co-expression of CD68, iNOS or MRC1/CD206, CD163, Arg1, and other markers in in vitro models. In human pathology, this subdivision is an oversimplification, and these markers can be expressed or downregulated in macrophages with high plasticity, according to different microenvironmental stimuli [18]. In Apc (Min/+) mice, the inhibition of PTGS2 reduces the M2 component [19]; thus, the expression of PTGS2 in CRC macrophages could be associated to the induction of a prevalent M2 phenotype. On the other hand, PGE2 is able to induce M1 differentiation in other mouse models [20], suggesting a positive influence of PTGS2 on the M1 component. We first tested the correspondence of PTGS2 expression with CD68 and CD163 on serial sections, assuming that CD68 positivity would indicate the total macrophage population, while CD163 would indicate the M2 component. The comparison of positive areas indicated a possible coexistence of PTGS2 and CD68 staining in some samples, while the correspondence of PTGS2 and CD163 was less evident (Figure 3a).

The quantification of cells, expressing these antigens in overlapping areas of equal extension, corroborated this observation (Figure 3b). The Pearson correlation coefficient was 0.422, (95% CI 0.229–0.582, p = 0.0000586) for CD68/PTGS2 and 0.316 (95% CI 0.110–0.496, p = 0.00324) for CD163/PTGS2.

**Figure 4:**
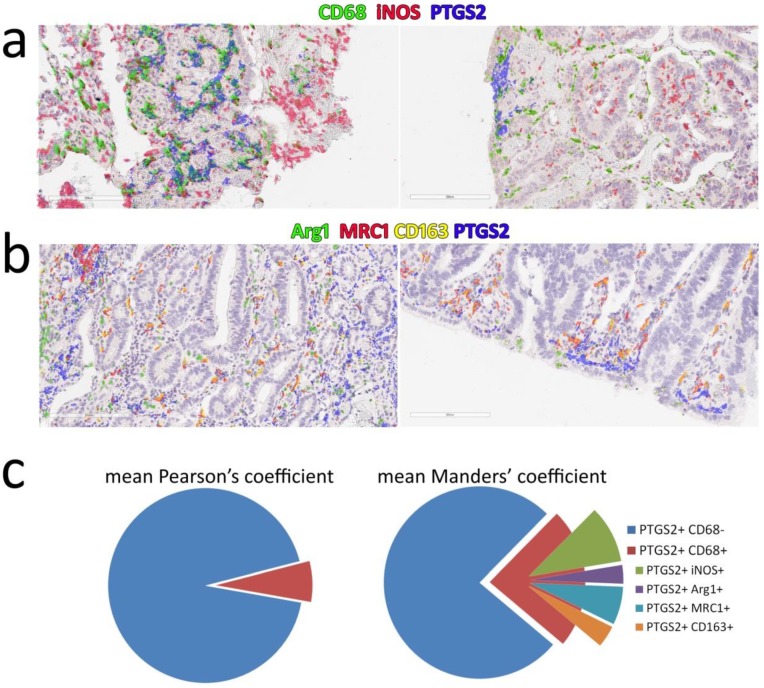
Multiplex IHC analysis of PTGS2 co-localization with macrophage markers CD68, iNOS, Arg1, MRC1/CD206, and CD163: (**a**) two examples of co-localization signals in the M1 series (CD68–iNOS–PTGS2), pseudocolors have been obtained overlaying the black&white mask of each marker on the first slide of the series; (**b**) two examples of co-localization signals in the M2 series (Arg1–MRC1–CD163–PTGS2); (**c**) left, mean Pearson’s coefficients of PTGS2 and CD68 co-localization; right: a simplified representation of the mean overlay between PTGS2 and each marker using Manders’ overlap coefficients. Slices indicate the extent of PTGS2 signal in the positive areas of each marker (complete analysis is shown in Supplementary Figure S2).

To obtain a more specific quantification of the involvement of M1 and M2 macrophages in PTGS2 production in CRC, we also tested a multiplex IHC approach. Using consecutive destaining, stripping, and reprobing of the same tissue slices, we tested the CD68–iNOS–PTGS2 and the Arg1–MRC1–CD163–PTGS2 series (Figure 4).

Multiplex analysis showed a complex picture with high variability among samples and different fields of the same sample. iNOS was strongly expressed by several non-macrophage cells, being the majority of iNOS positive signals in CRC tissues (Figure 4a). Few Arg1^dim^-positive cells were detected, with minimal overlap with other M2 markers (Figure 4b and Supplementary Figure S1). A strong overlay was observed only between MRC1 and CD163 (Figure 4b), which was confirmed by co-localization analysis (Supplementary Figure S2). PTGS2 showed a limited co-localization with macrophages: the mean Pearson’s coefficient for CD68-PTGS2 was 0.063, while the mean Manders’ overlap coefficient, evaluating the extent of PTGS2 positivity in the CD68-positive area, was 0.237 (Figure 4c and Supplementary Figure S2). We also attempted a simplified representation of the co-localization of M1 and M2 single markers, assuming their expression only in macrophages (Figure 4c, right). This analysis suggested a mixed contribution of both M1 and M2 polarized cells in PTGS2 expression, with a possible prevalence of iNOS+ cells.

Thus, the expression of PTGS2 at the luminal surface of CRC, in non-tumor cells, did not apparently associate to a M1->M2 switch of macrophages. Moreover, macrophages did not appear as the main PTGS2-positive cell population in stromal areas.

**Figure 5:**
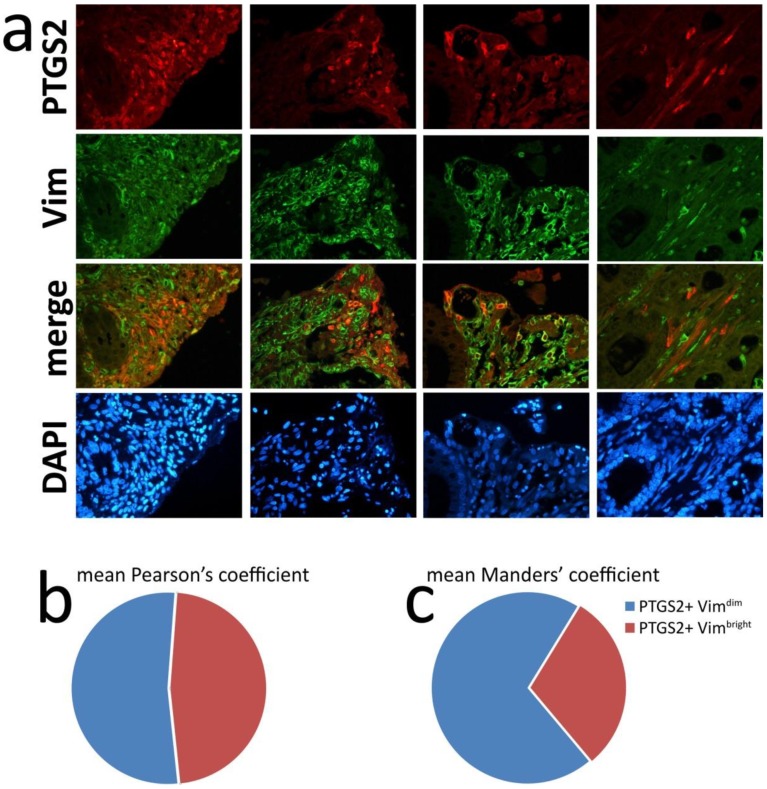
Immunofluorescent double staining of PTGS2 and vimentin indicates cancer-associated fibroblasts as a major source of PTGS2: (**a**) Representative images of PTGS2 and vimentin double immunofluorescence in four CRC. PTGS2 positivity almost invariably corresponded to vimentin positive cells, although bright PTGS2 staining frequently corresponded to dim vimentin staining and vice versa; (**b**) mean PTGS2–vimentin co-localization evaluated by Pearson’s coefficient after the subtraction of dim vimentin positivity; (**c**) mean Manders’ overlap coefficient evaluating the extent of PTGS2 signal in bright vimentin-positive areas (complete analysis is shown in Supplementary Figure S3).

Adegboyega et al. described subepithelial myofibroblasts as another source of PTGS2 in colorectal adenomas, mainly in luminal mucosal areas with damaged surface [21]. Sonoshita et al. observed the same localization of PTGS2 in the Apc^Δ716^ mouse model and in human adenomas, finding a strong co-localization of PTGS2 and vimentin, the marker of mesenchymal cells [22]. To verify the involvement of mesenchymal cells, we performed fluorescent double staining to co-localize PTGS2 and vimentin in multiple fields of representative CRC samples (Figure 5a).

Indeed, almost all PTGS2-positive cells corresponded to vimentin-positive cells, although stromal cells with the bright PTGS2 signal usually showed a lower signal for vimentin. Eliminating dim vimentin-positive signals by a color threshold, co-localization analysis showed a mean Pearson co-localization coefficient of 0.472 (Figure 5b; range 0.367–0.600, see Supplementary Figure S3a). Manders’ overlap coefficients, evaluating the overlap of PTGS2 with vimentin and vice versa, showed that a high proportion of vimentin-positive PTGS2-negative mesenchymal cells was also present (Supplementary Figure S3b). The mean of Manders’ overlap coefficients evaluating the extent of PTGS2 signal in bright vimentin-positive areas was 0.301 (Figure 5c). The possible involvement of cancer-associated fibroblasts (CAF) as a prevalent non-tumor source of PTGS2 in CRC led us to analyze in vitro the possible inducers of PTGS2 in primary colorectal CAF.

### 3.3. In Vitro, IL1β is a Powerful Inducers of PTGS2 in CAF and Correlates to gPTGS2 Levels in Tissue Lysates

**Figure 6:**
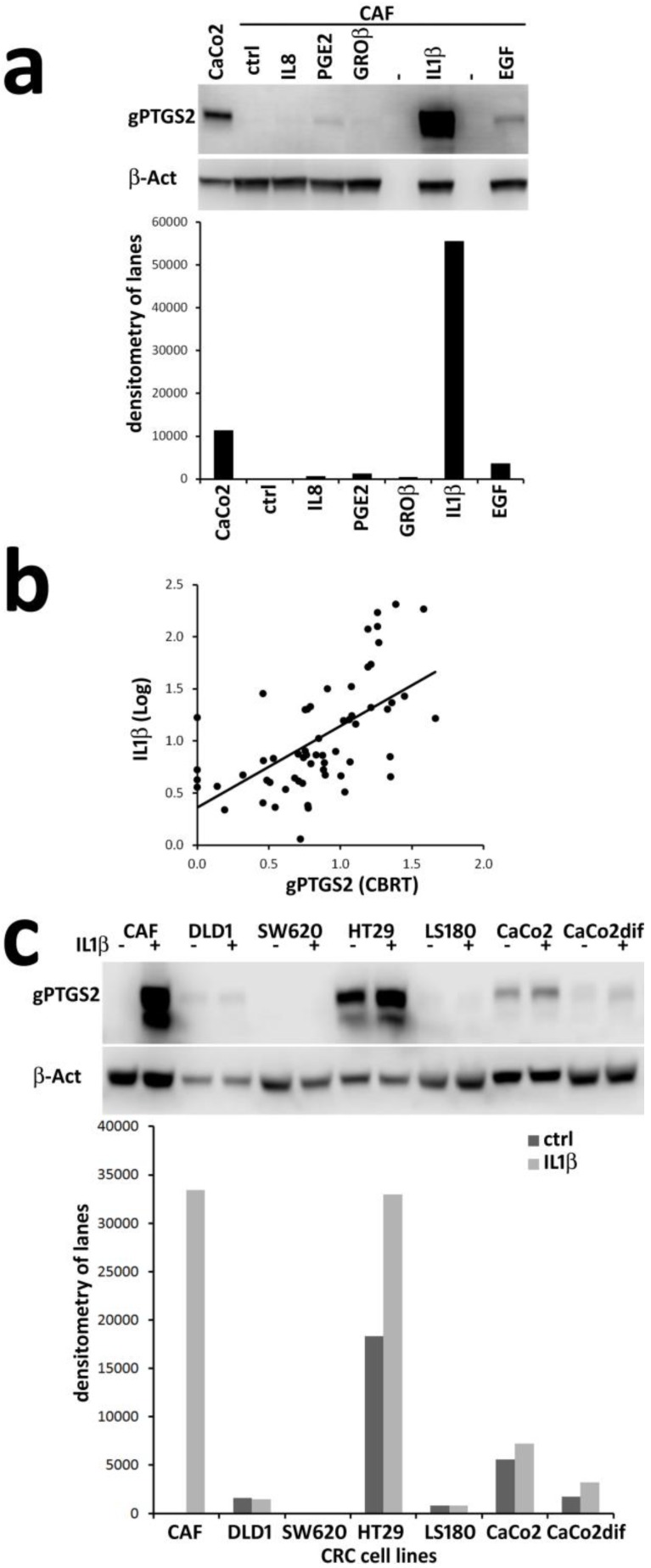
IL1β strongly upregulates PTGS2 expression in cancer-associated fibroblasts (CAF) and correlates to gPTGS2 levels in tissue lysates: (**a**) The well-known PTGS2 inducer IL1β and other four cytokines involved in CRC (CXCL8/IL8, PGE2, CXCL2/GROβ, and EGF; 24 h treatment) were tested as possible inducers of PTGS2 expression in primary CRC CAF. Only IL1β induced a powerful response. Densitometric quantifications of WB lanes are plotted; (**b**) Linear regression of PTGS2 and IL1β levels in CRC tissue lysates (60 samples). Pearson’s correlation coefficient of gPTGS2 (CBRT = cubic root) vs. IL1β (Log) levels was 0.593 (p = 0.00000609; (**c**) IL1β (24 h treatment) has a reduced ability to modify PTGS2 basal expression in CRC cell lines. Densitometric quantifications of Western blot lanes are plotted. WB were run as experimental duplicates, with similar results (see Supplementary Figure S4).

CAF primary cells [15] were stimulated for 24 h with IL1β, a known inducer of PTGS2 expression by NFkB and AP1 activation [23]. Other factors linked to inflammatory/trophic CRC progression (IL8/CXCL8, GROβ/CXCL2, as agonists of G-protein coupled receptors; PGE2 produced by PTGS2 activation; EGF as the prototype EGFR tyrosine-kinase agonist) were also tested as potential PTGS2 inducers [1,24,25,26]. The analysis for gPTGS2 expression by Western blot showed a powerful induction of gPTGS2 by IL1β, and weak responses elicited by PGE2 and EGF (Figure 6a).

IL1β involvement in the induction of gPTGS2 in our cohort was verified by the quantification of its levels in 60 tissue lysates (Figure 6b). Pearson correlation coefficient of gPTGS2 versus IL1β levels was 0.593 (p = 0.00000609), indicating a strong link between IL1β and gPGTS2 levels in more than half of the samples. Western blot analysis of CRC cell lines stimulated by IL1β revealed a possible explanation for the existence of samples with a low gPTGS2/IL1β correlation. CRC cell lines showed high or low basal levels of gPTGS2 with a limited response to IL1β stimulation compared to CAF (Figure 6c), even at 10× increased doses of IL1β (Supplementary Figure S4). Thus, the prevalence of stroma, or tumor-derived gPTGS2, could mirror the strong or weak response to the presence of IL1β in CRC tissues.

### 3.4. Effects of Stromal PTGS2 on Patients Prognosis

**Figure 7:**
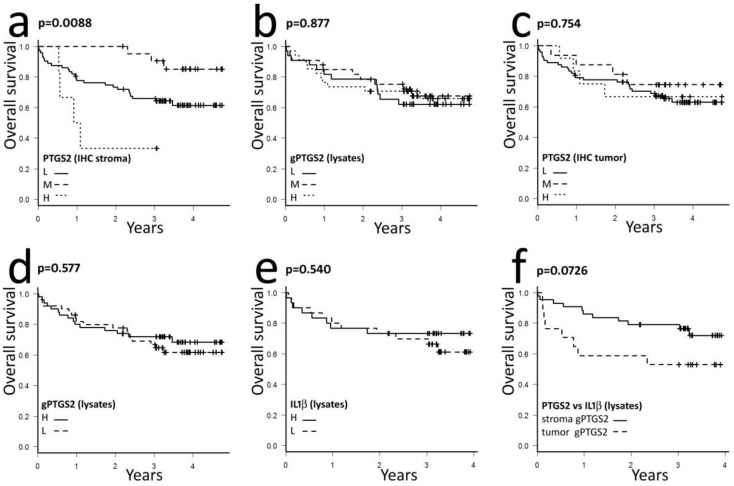
Effects of PTGS2 localization on patients overall survival: (**a**) Kaplan–Meier analysis of patients’ overall survival according to PTGS2 levels quantified by IHC in stromal cells. L = low, M = medium, or H = high PTGS2 expression; (**b**) Kaplan–Meier analysis of patients’ overall survival according to gPTGS2 levels quantified in tissue lysates by WB. L=low, M=medium, or H=high gPTGS2 expression; (**c**) Kaplan–Meier analysis of patients overall survival according to PTGS2 levels quantified by IHC in tumor epithelial cells. L=low, M=medium, or H=high PTGS2 expression: (**d–e**) gPTGS2 and IL1β levels detected in tissue lysates by WB were dichotomized (low vs. high) using the median value of data as a threshold. Kaplan–Meier analysis of patients’ overall survival against low or high gPTGS2 (d) or IL1β (e) levels was not affected by this dichotomization; (**f**) Dichotomization shown in panels d–e was used to separate tumors into two new classes: the first with a directly proportional regulation of gPTGS2 and IL1β (gPTGS2^low^ILβ^low^ + gPTGS2^high^ILβ^high^ = stroma gPTGS2 enriched, continuous line), the other with an independent regulation of these markers (gPTGS2^low^ILβ^high^ + gPTGS2^high^ILβ^low^ = tumor gPTGS2 enriched, dashed line). The Kaplan–Meier analysis of patients’ overall survival based on this subdivision is shown.

In our cohort, stromal PTGS2 affected patients OS according to low, medium, or high IHC positivity (Figure 7a). In particular, intermediate levels of stromal PTGS2 (n = 22) were apparently associated to a better prognosis, while high PTGS2 (n = 6) showed a negative outcome compared to low/null PTGS2-expressing CRC.

The exclusion of stage IV tumors from Kaplan–Meier analysis did not modify the prognostic potential of stromal PTGS2 expression (Supplementary Figure S5), reducing a potential bias on OS evaluation linked to the surgical or therapeutic treatment of this group of patients. Neither gPTGS2 total levels quantified in tissue lysates nor tumor-associated PTGS2 scored by IHC influenced patients OS in our cohort (Figure 7b,c).

Our observations suggested a possible subgrouping of CRC on the basis of stromal, or tumor-derived PTGS2, the former modulated by IL1β, with a prognostic significance, the latter relatively independent. We asked if we could identify these two categories of tumors on the basis of gPTGS2 and IL1β levels in tissue lysates and if they could influence patients prognosis. When dichotomized by the median value, PTGS2 showed no relation with the main pathological data (Table 1), but it maintained a significant relation with IL1β (the database with pathological data, PTGS2, and IL1β quantification is available in Supplementary data S1).

We used gPTGS2 and IL1β dichotomization by the median of data and defined as stroma-derived gPTGS2 the cases with low + low or high + high gPTGS2 and IL1β levels (= directly proportional IL1β and gPTGS2 levels). On the contrary, tumor-derived gPTGS2 was defined in cases with low + high or high + low gPTGS2 and IL1β levels (= independence of IL1β and gPTGS2 expression). Dichotomized gPTGS2 or IL1β levels, individually assessed by Kaplan–Meier analysis, did not influence patients’ OS, showing almost superimposable curves (Figure 7d,e). On the contrary, an immediate divergence of curves was observed when CRC with putative stroma-derived PTGS2 were compared to CRC with putative tumor-derived PTGS2 (Figure 7f). This categorization suggested a different involvement of stroma-expressed versus tumor-expressed PTGS2 in patient outcomes, although it did not reach statistical significance.

## 4. Discussion

Several epidemiological studies suggested a favorable outcome for patients taking NSAIDS after CRC diagnosis/surgery [5,6,7]. An ideal approach for PTGS2 targeting would be the identification of CRC patients that could benefit from the inclusion of PTGS2 inhibitors in the adjuvant setting. The influence of PTGS2 expression on patient prognosis could be a possible criterion, but the quantification of PTGS2 by IHC only in CRC tumor cells showed variable results [8,9,10,11]. We attempted to overcome this limitation quantifying gPTGS2 levels in CRC lysates, comparing them with IHC-scored, tumor, or stroma-derived PTGS2. 

Our approach shows that gPTGS2 levels can be quantified in CRC lysates with high sensitivity and specificity. gPTGS2 levels partially correlated with both tumor-associated and stroma-associated PTGS2 detected by IHC, indicating that gPTGS2 expression is not restricted to tumor cells as hypothesized before. Stroma-associated PTGS2 showed an almost exclusive luminal distribution, as already observed in adenomas [21,22], suggesting a homeostatic role in preserving the mucosal barrier. Macrophages did not appear as a major PTGS2-positive cell population, although minor populations of macrophages expressing M1 (iNOS) or M2 (MRC1, CD163) markers co-localized with the PTGS2 signal. The slight prevalence of the PTGS2 signal in iNOS-positive macrophages would be in accordance with the preferential expression of PTGS2 by M1-polarized macrophages observed in vitro [27]. Multiplex IHC analysis showed that only MRC1 and CD163 were frequently associated, while Arg1 expression was rare and iNOS was expressed by several different cells without relation to CD68. While macrophages could directly contribute to PTGS2 expression in CRC, their specific ability to produce IL1β [28] would be a major mechanism of PTGS2 amplification by the paracrine stimulation of bystander fibroblasts. Indeed, Cui et al. showed that most IL1β-expressing cells localize in the stroma of CRC (median 19.2 cells/high power field, hpf), while positive epithelial tumor cells are rare (median 0.4 cells/hpf) [29]. Our in vitro data and CRC lysates analysis sustain the hypothesis that gPTGS2 expression could be mediated by IL1β, preferentially targeting CAF. Accordingly, Cui et al. showed a higher positivity for IL1R1 in the stroma (median 11.0 cells/hpf) than in tumor epithelial cells (median 0.7 cells/hpf) [29]. IL1β is neo-synthesized and activated by the inflammasome only in the presence of microbial components or tissue damage [28], sustaining the plausible role of luminal, stromal PTGS2 for the homeostatic rescue of the epithelial barrier. Indeed, several bacterial species have been associated to CRC [30], and *Streptococcus gallolyticus* can infect colorectal tumors and has been linked to local IL1β and PTGS2 expression [31]. According to these data, stromal PTGS2 expressed in the luminal area could exert a protective role for the patient, not necessarily influencing CRC progression. Unfortunately, we could not evaluate this hypothesis as only overall survival data were available for our cohort, without information about the cause of death.

Tumor-associated PTGS2 did not apparently affect patients’ overall survival, and the quantification of specific gPTGS2 levels in tissue lysates could not discriminate patient outcome. Tumor epithelial PTGS2 could be less controlled by physiologic stimuli as some oncogenic mechanisms can affect its expression; for example, PTGS2 is frequently downregulated in MSI CRC, while PIK3CA mutation could mediate PTGS2 activity [32,33,34]. When gPTGS2 and IL1β levels were used to discriminate those CRC depending on IL1β for gPTGS2 expression (enriched in stromal gPTGS2) from tumors with an independent gPTGS2 expression (enriched in tumor gPTGS2), survival curves showed an immediate dichotomy. While this datum has no significant prognostic implications, it suggests distinct roles for stromal and tumor PTGS2.

## 5. Conclusion

Our study suggests an unpredicted association of stromal PTGS2 with patients’ prognosis that limits the use of total gPTGS2 quantification in CRC samples lysates for predictive purposes. Due to the possible positive influence on patient OS of an intermediate PTGS2 expression in the luminal tumor stroma, we propose further validation of this marker on larger cohorts.

## Figures and Tables

**Table 1:** Clinical and molecular features of CRC according to median PTGS2 levels.

Clinicopathologic Parameters	All	PTGS2 < Median	PTGS2 > Median	*p*
***TOTAL***	**100**	**50**	**50**	-
***SEX***				*0.689*
Male	**49**	26	23	
Female	**51**	24	27	
***AGE (surgery)***				*0.317*
≤70	**47**	22	28	
>70	**53**	28	22	
TUMOR LOCATION *				*0.965*
Proximal colon	**43**	21	22	
Distal colon	**36**	18	18	
Rectum	**21**	11	10	
***STAGE (UICC-2009)***				*0.974*
I	**14**	7	7	
II	**34**	16	18	
III	**39**	20	19	
IV	**13**	7	6	
***TUMOR GRADE***				*0.479*
well	**5**	2	4	
moderate	**75**	40	35	
poor	**19**	8	11	
***JASS SCORE***				*0.719*
I	**17**	7	10	
II	**14**	6	8	
III	**32**	18	14	
IV	**37**	19	18	
***PERINEURAL INVASION***				*0.387*
PNI 0	**69**	37	32	
PNI 1	**31**	13	18	
MICROSAT. INSTAB. **				*0.158*
MSS	**72**	40	32	
MSI	**15**	5	10	
IL1β ***				*0.002*
IL1β<median	**30**	21	8	
IL1β>median	**30**	10	22	

* Proximal colon includes cecum to transverse colon; distal colon includes splenic flexure to sigmoid colon. ** Data available for 87 samples. *** Data available for 60 samples. NOTE: 2 × 2 tables were tested by Fisher’s exact test, other parameters by Chi-square test.

## Supplementary Materials

The following are available online at https://www.mdpi.com/2073-4409/9/3/683/s1, Table S1: PTGS2/COX2 expression in CRC (literature); Supplementary method S1: anti-PTGS2 antibody selection; Figure S1: Example of multiplex IHC and method for overlay computation; Figure S2: Analysis of 1:1 colocalization of M1 and M2 macrophage markers with PTGS2 by the JACoP plug-in; Figure S3: JACop colocalization analysis of PTGS2 and Vimentin fluorescent staining; Figure S4: Confirm of Western blot data shown in Figure 6; Figure S5: Effects of PTGS2 localization on patients overall survival (stage IV CRC excluded); Supplemetary data S1: database with pathological data and PTGS2 and IL1β quantification (excel file).
