# Supplementary material for: Evaluation of Glycosylated PTGS2 in Colorectal Cancer for NSAIDS-Based Adjuvant Therapy
Source: Cells. 2020 Mar 11;9(3):683. doi: 10.3390/cells9030683 (PMC7140631; doi:10.3390/cells9030683)
Supplement: Supplementary file 1 [file cells-09-00683-s001.zip › Suppl files/Supplementary Figure S2.pdf]

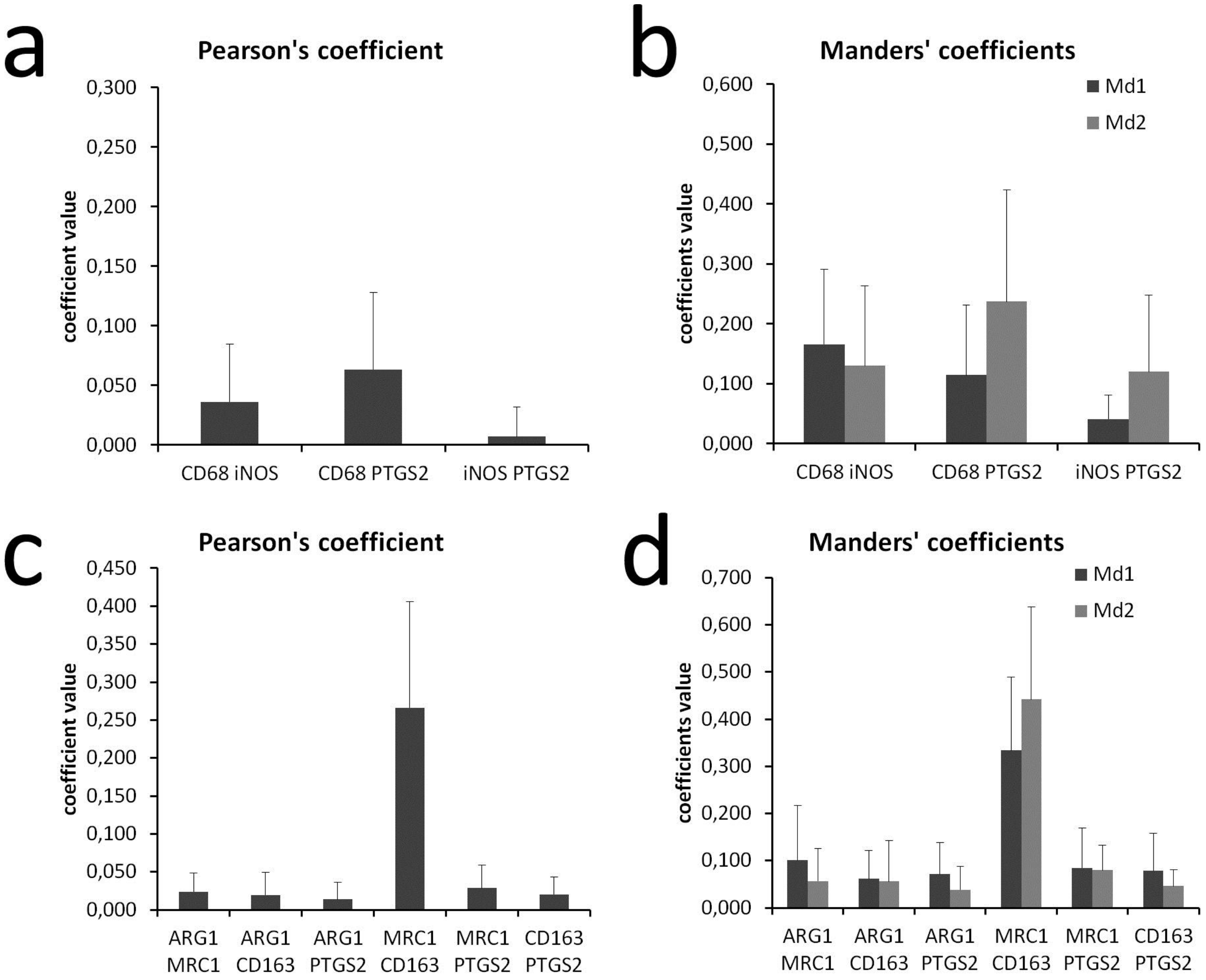

Supplementary Figure S2. Analysis of 1:1 colocalization of M1 and M2 macrophage markers with PTGS2 by the JACoP plug-in: mean and standard deviation of Pearson's correlation coefficients in the M1 (**a**) and M2 (**c**) series; mean and standard deviation of Manders' overlap coefficients in the M1 (**b**) and M2 (**d**) series.

Note: Manders' overlap coefficients express the extent of co-occurrence of two markers. Md1 and Md2 represent the colocalization of the first marker over the second and vice versa. For example in panel b the CD68 - PTGS2 relation is evaluated as Md1 (CD68 signal overlapping with PTGS2) and Md2 (PTGS2 signal overlapping with CD68) and shows a prevalence of the second overlap.
