# Supplementary material for: Evaluation of Glycosylated PTGS2 in Colorectal Cancer for NSAIDS-Based Adjuvant Therapy
Source: Cells. 2020 Mar 11;9(3):683. doi: 10.3390/cells9030683 (PMC7140631; doi:10.3390/cells9030683)
Supplement: Supplementary file 1 [file cells-09-00683-s001.zip › Suppl files/Supplementary Figure S3.pdf]

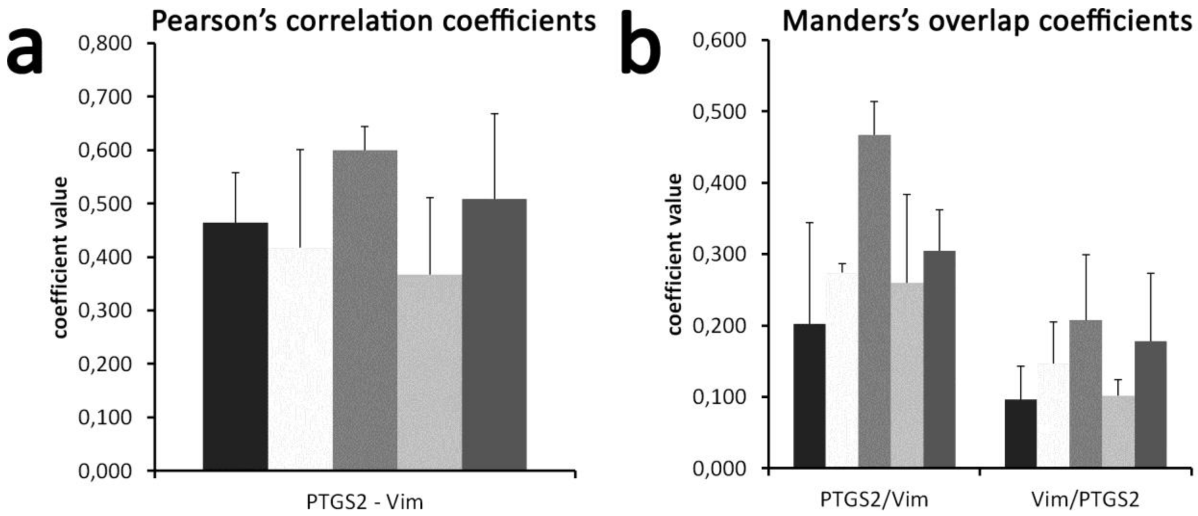

Supplementary Figure S3. JACop colocalization analysis of PTGS2 and Vimentin fluorescent staining: graphical representation of Pearson's (**a**) and Manders' (b) analyses of PTGS2 and vimentin colocalization in multiple fields of five CRC cases, after threshold subtraction of dim vimentin -positive cells. Mean Pearson correlation index ranged from 0.367 to 0.600 indicating a high degree of PTGS2-vimentin colocalization also after eliminating a potential spurious signal. Manders overlap coefficients confirm that PTGS2 has a high correspondence with vimentin, while the opposite linkage is less evident.
