# Supplementary material for: Evaluation of Glycosylated PTGS2 in Colorectal Cancer for NSAIDS-Based Adjuvant Therapy
Source: Cells. 2020 Mar 11;9(3):683. doi: 10.3390/cells9030683 (PMC7140631; doi:10.3390/cells9030683)
Supplement: Supplementary file 1 [file cells-09-00683-s001.zip › Suppl files/Supplementary Figure S4.pdf]

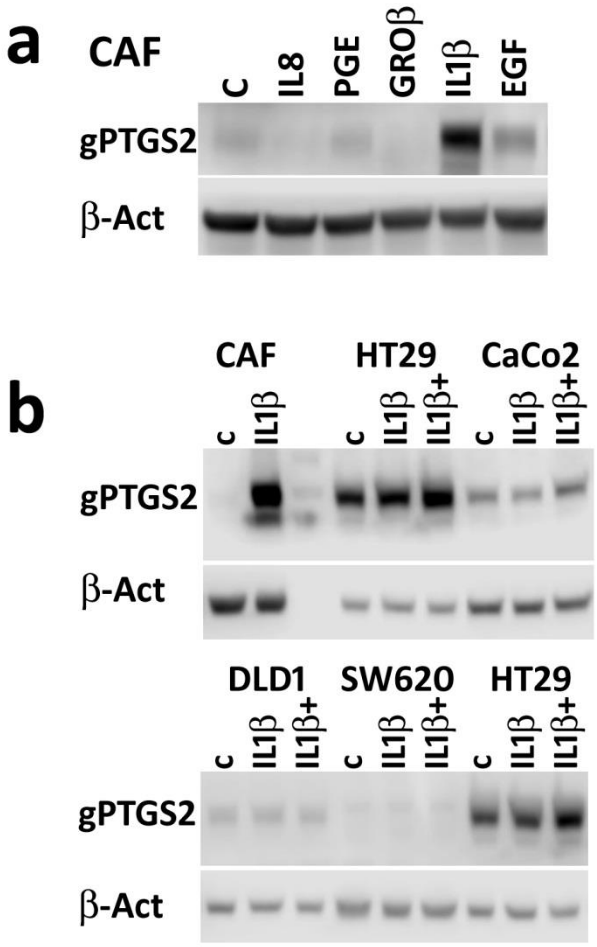


**Supplementary Figure S2.** Confirm of western blot data shown in Figure 4: (**a**) induction of PTGS2 expression in CAF primary cultures by CXCL8/IL8 (10 ng/ml), PGE2 (100 nM), CXCL2/Groβ (10 ng/ml), IL1β (0.1 ng/ml), EGF (10ng/ml), 24h treatment; (**b**) CAF and CRC cell lines treated with 0.1ng/ml (IL1β) or 1ng/ml (IL1β+) IL1β. A 10x IL1β increase substantially affected PTGS2 levels only in the HT29 cell line.
