# Supplementary material for: Evaluation of Glycosylated PTGS2 in Colorectal Cancer for NSAIDS-Based Adjuvant Therapy
Source: Cells. 2020 Mar 11;9(3):683. doi: 10.3390/cells9030683 (PMC7140631; doi:10.3390/cells9030683)
Supplement: Supplementary file 1 [file cells-09-00683-s001.zip › Suppl files/Supplementary Figure S5.pdf]

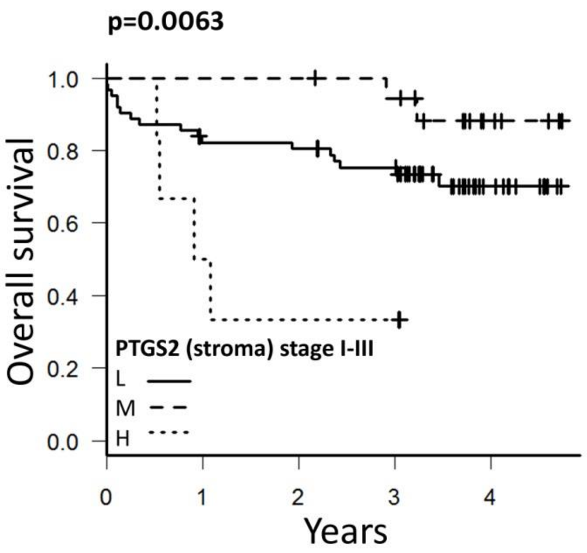

Supplementary Figure S3. Effects of PTGS2 localization on patients overall survival: Kaplan-Meier analysis of patients overall survival (stage I-III CRC only) according to PTGS2 levels quantified by IHC in stromal cells. L=low, M=medium, or H=high PTGS2 expression. The exclusion of stage IV patients does not modify the prognostic prediction of stromal PTGS2 expression, excluding a possible bias in OS evaluation linked to the persistence or resection of hepatic metastases..
