# Supplementary material for: Evaluation of Glycosylated PTGS2 in Colorectal Cancer for NSAIDS-Based Adjuvant Therapy
Source: Cells. 2020 Mar 11;9(3):683. doi: 10.3390/cells9030683 (PMC7140631; doi:10.3390/cells9030683)
Supplement: Supplementary file 1 [file cells-09-00683-s001.zip › Suppl files/Supplementary method S1.pdf]

Selection of PTGS2/COX-2 antibody and detection efficiency in CRC samples.

Antibody selection.

Based on IHC of gastric adenocarcinoma, Saukkonen et al previously selected the Cayman mAb 160112 as the best anti-PTGS2 antibody among seven products tested (160106, 160107, 160112, 160116, 160126 from Cayman; PG 27 from Oxford Biomedical Research; sc-1745 from Santa Cruz Biotechnology) [1]. This antibody was also used in three studies detecting PTGS2 in large cohorts of CRC patients (see Suppl Tab1). On the other hand Adegboyega [2] identified by WB (on C18 fibroblasts) Cayman affinity purified pAb 160126 as the most specific anti PTGS2 antibody among those tested (160106, 160107, 160112, 160126 from Cayman; sc1745, sc1746, sc7951 from Santa Cruz Biotechnology; 610203 from DB).

Starting from these observations, we compared in WB the 160112 mouse mAb and 160126 rabbit pAb Cayman antibodies, to a relatively new product from Cell Signaling, the D5H5 rabbit mAb. This antibody was raised against a synthetic peptide corresponding to residues surrounding His108 of human PTGS2, far from the carboxy-terminus targeted by Cayman antibodies. First, we tested these antibodies on CRC cell lines known to express (CaCo2, HT29) or not (HCT15, SW480) PTGS2 [3], and on a standard of ovine PTGS2 (Figure A1a). In this experimental setting, 160126 showed less sensitivity and specificity as compared to the other antibodies, and was excluded from further testing. In the second step of selection D5H5 and 160112 were tested on the paired lysates from normal mucosa (N) and tumor tissue (K) of five CRC patients (Figure A1b). 160112 antibody revealed several spurious bands next to the expected molecular weight of PTGS2, not allowing an accurate densitometry of the 72kDa glycosylated form. A stronger and more specific signal was detected by D5H5 antibody, that was chosen as the most suitable tool to analyze PTGS2 levels in CRC lysates by WB.

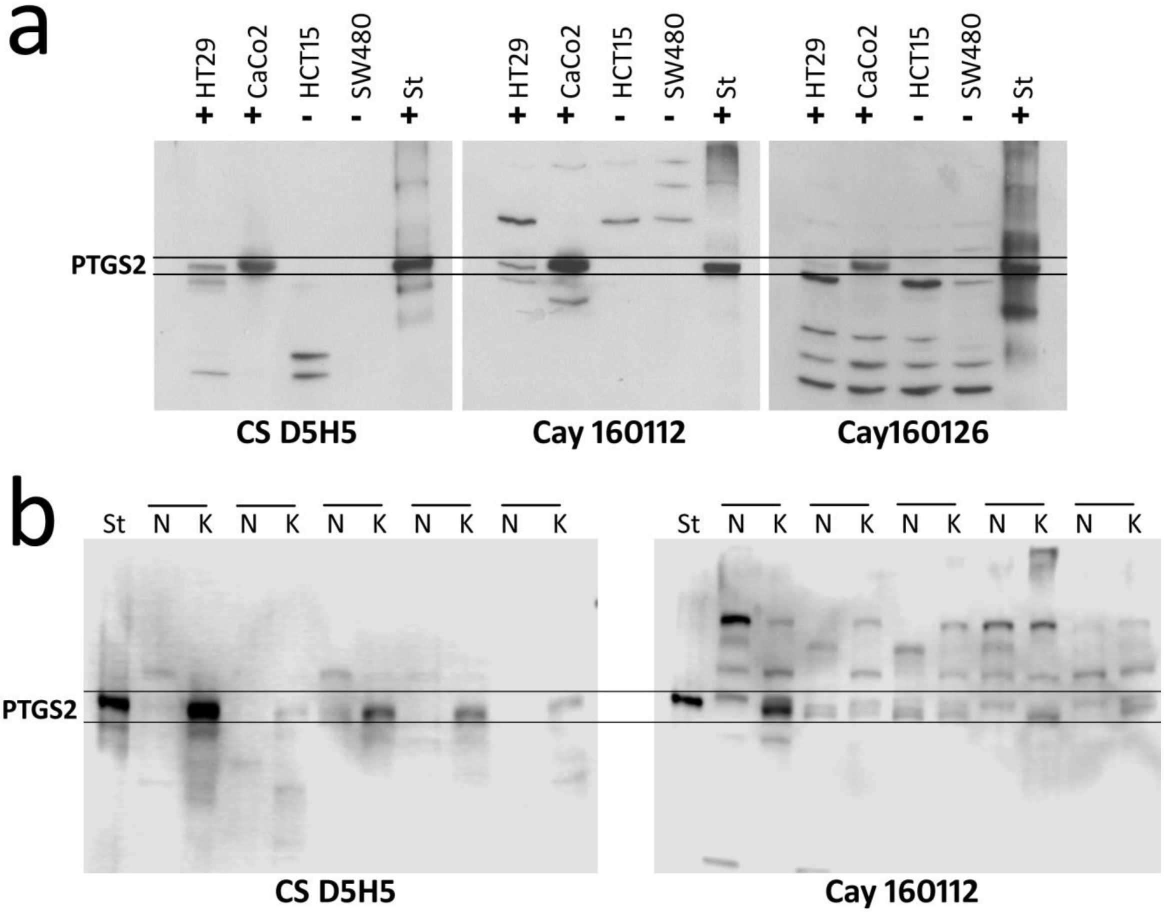

Figure A1. Selection of the anti-PTGS2 antibody for WB detection: (**a**) Three candidate antibodies (D5H5 from Cell Signaling Technologies, 160112 and 160126 from Cayman Chemicals) were tested on the lysates of colon cancer cell lines known to express (HT29, CaCo2) or not (HCT15, SW480) PTGS2; ovine PTGS2 from microsomial fractioning was used as standard (St); (**b**) D5H5 and 160112 antibodies, selected according to panel (a) results, were tested on lysates from the normal mucosa (N) and the corresponding CRC tissue (K) of five patients.

References.

1. Saukkonen, K.; Nieminen, O.; van Rees, B.; Vilkki, S.; Härkönen, M.; Juhola, M.; Mecklin, J.P.; Sipponen, P.; Ristimäki, A. Expression of cyclooxygenase-2 in dysplasia of the stomach and in intestinal-type gastric adenocarcinoma. *Clin. Cancer Res. Off. J. Am. Assoc. Cancer Res.* **2001**, *7*, 1923–1931.
2. Adegboyega, P.A.; Ololade, O.; Saada, J.; Mifflin, R.; Di Mari, J.F.; Powell, D.W. Subepithelial myofibroblasts express cyclooxygenase-2 in colorectal tubular adenomas. *Clin. Cancer Res. Off. J. Am. Assoc. Cancer Res.* **2004**, *10*, 5870–5879.
3. Dommels, Y.E.M.; Haring, M.M.G.; Keestra, N.G.M.; Alink, G.M.; van Bladeren, P.J.; van Ommen, B. The role of cyclooxygenase in n-6 and n-3 polyunsaturated fatty acid mediated effects on cell proliferation, PGE(2) synthesis and cytotoxicity in human colorectal carcinoma cell lines. *Carcinogenesis* **2003**, *24*, 385–392.
