# Supplementary material for: Evaluation of Glycosylated PTGS2 in Colorectal Cancer for NSAIDS-Based Adjuvant Therapy
Source: Cells. 2020 Mar 11;9(3):683. doi: 10.3390/cells9030683 (PMC7140631; doi:10.3390/cells9030683)
Supplement: Supplementary file 1 [file cells-09-00683-s001.zip › Suppl files/Supplementary Table S1.pdf]

<u>Supplementary Table 1</u>

PTGS2/COX2 expression in CRC (literature)

Reference	n° patients	Mucosa	CRC	Method	Antibody	Immunogen	Source
**[1]**	37 (25 CRC)	8%	76%	WB	rabbit antiserum	full ovine PTGS2	Home - made
**[2]**	39	not reported	56,4%<br>(90% Rect, 21% Col)	WB	nd	hu PTGS2, region not specified	Santa Cruz
**[3]**	30	23%	73%	WB	ab1519<br>rabbit polyclonal	carboxy-terminal peptide (rat), sequence not available	Abcam
**[4]**	31	negative (IHC)<br>20% (WB 3/15)	96,7% (IHC 30/31)<br>93% (WB 14/15)	IHC<br>WB	SC-1745 (IHC)<br>goat polyclonal<br>nd (WB)<br>polyclonal	carboxy-terminal peptide (hu), sequence not available	Santa Cruz (IHC)<br>Cayman (WB)
**[5]**	35 (31 analyzed)	nd	87%	IHC	160106<br>rabbit polyclonal	AA 570-598 (murine)	Cayman
**[6]**	76	nd	81,5%	IHC	rabbit antiserum	AA 588-600 (hu)	Home- made
**[7]**	112	17%	72%	IHC	nd, rabbit polyclonal<br>(*SC-7951?* †)	AA 50-111 hu	Cayman<br>(*Santa Cruz?* †)
**[8]**	170 (139 CRC)	24,1% (7/29)	84,9%	IHC	nd	nd	Cayman
**[9]**	288	10% (1/10)	70,8% (204/288)	IHC	nd	nd	Cayman
**[10]**	747	nd	85,3%	IHC	SC-1745<br>goat polyclonal	carboxy-terminal peptide (hu), sequence not available	Santa Cruz
**[11]**	167 (23 CRC)	negative	40%	IHC	clone CX-294<br>mouse mAb	AA 580-598 hu	Dako
**[12]**	636	nd	67%	IHC	160112 (‡)<br>mouse mAb	AA 580-599 hu	Cayman
**[13]**	758 (207 CRC)	nd	85%<br>(65% strong)	IHC	160112 (‡)<br>mouse mAb	AA 580-599 hu	Cayman
**[14]**	662	nd	83%	IHC	160112 (‡)<br>mouse mAb	AA 580-599 hu	Cayman
**[15]**	118 (Col)<br>85 (Rect)	nd	93% (Col)<br>87% (Rect)	IHC	SP21 rabbit mAb	carboxy-terminal peptide (rat), sequence not available	Thermo Fisher Scientific
**[16]**	1026	12% (6/50)	77.9% (800/1026)	IHC	SP21 rabbit mAb	carboxy-terminal peptide (rat), sequence not available	Zhongshan Golden Bridge Biotechnology

† At present the only PTGS2 antibody raised against AA 50-111 is SC-7951 (Santa Cruz), we do not know if a similar product was available from Cayman in 2002.

‡ These papers used the same Cayman antibody, but we were unable to categorize it. Prof. Ogino has kindly provided us the exact product identity.

References:

1. Kargman SL, O'Neill GP, Vickers PJ, Evans JF, Mancini JA and Jothy S: Expression of Prostaglandin G/H Synthase-1 and -2 Protein in Human Colon Cancer. Cancer Res 55: 2556–2559, 1995.
2. Dimberg J, Samuelsson A, Hugander A and Soderkvist P: Differential expression of cyclooxygenase 2 in human colorectal cancer. Gut 45: 730–732, 1999.
3. Asting AG, Farivar A, Iresjö B-M, Svensson H, Gustavsson B and Lundholm K: EGF receptor and COX-1/COX-2 enzyme proteins as related to corresponding mRNAs in human per-operative biopsies of colorectal cancer. BMC Cancer 13: 511, 2013.
4. Cianchi F, Cortesini C, Bechi P, *et al.*: Up-regulation of cyclooxygenase 2 gene expression correlates with tumor angiogenesis in human colorectal cancer. Gastroenterology 121: 1339–1347, 2001.
5. Shattuck-Brandt RL, Lamps LW, Heppner Goss KJ, DuBois RN and Matrisian LM: Differential expression of

matrilysin and cyclooxygenase-2 in intestinal and colorectal neoplasms. Mol Carcinog 24: 177–187, 1999.
6. Sheehan KM, Gulmann C, Eichler GS, *et al.*: Signal pathway profiling of epithelial and stromal compartments of colonic carcinoma reveals epithelial-mesenchymal transition. Oncogene 27: 323–331, 2008.
7. Zhang H and Sun X-F: Overexpression of cyclooxygenase-2 correlates with advanced stages of colorectal cancer. Am J Gastroenterol 97: 1037–1041, 2002.
8. Wu A-W, Gu J, Ji J-F, Li Z-F and Xu G-W: Role of COX-2 in carcinogenesis of colorectal cancer and its relationship with tumor biological characteristics and patients' prognosis. World J Gastroenterol 9: 1990–1994, 2003.
9. Soumaoro LT, Uetake H, Higuchi T, Takagi Y, Enomoto M and Sugihara K: Cyclooxygenase-2 expression: a significant prognostic indicator for patients with colorectal cancer. Clin Cancer Res Off J Am Assoc Cancer Res 10: 8465–8471, 2004.
10. Fux R, Schwab M, Thon K-P, Gleiter CH and Fritz P: Cyclooxygenase-2 Expression in Human Colorectal Cancer Is Unrelated to Overall Patient Survival. Clin Cancer Res 11: 4754–4760, 2005.
11. Balbinotti RA, Ribeiro U, Sakai P, *et al.*: hMLH1, hMSH2 and cyclooxygenase-2 (cox-2) in sporadic colorectal polyps. Anticancer Res 27: 4465–4471, 2007.
12. Chan AT, Ogino S and Fuchs CS: Aspirin and the Risk of Colorectal Cancer in Relation to the Expression of COX-2. N Engl J Med 356: 2131–2142, 2007.
13. Kawasaki T, Nosho K, Ohnishi M, *et al.*: Cyclooxygenase-2 overexpression is common in serrated and non-serrated colorectal adenoma, but uncommon in hyperplastic polyp and sessile serrated polyp/adenoma. BMC Cancer 8: 33, 2008.
14. Ogino S, Kirkner GJ, Nosho K, *et al.*: Cyclooxygenase-2 expression is an independent predictor of poor prognosis in colon cancer. Clin Cancer Res Off J Am Assoc Cancer Res 14: 8221–8227, 2008.
15. Lobo Prabhu KC, Vu L, Chan SK, Phang T, Gown A, Jones SJ and Wiseman SM: Predictive utility of cyclo-oxygenase-2 expression by colon and rectal cancer. Am J Surg 207: 712–716, 2014.
16. Wu Q-B and Sun G-P: Expression of COX-2 and HER-2 in colorectal cancer and their correlation. World J Gastroenterol 21: 6206–6214, 2015.
