# Supplementary figures and images for: Evaluation of Glycosylated PTGS2 in Colorectal Cancer for NSAIDS-Based Adjuvant Therapy
Source: Cells. 2020 Mar 11;9(3):683. doi: 10.3390/cells9030683 (PMC7140631; doi:10.3390/cells9030683)

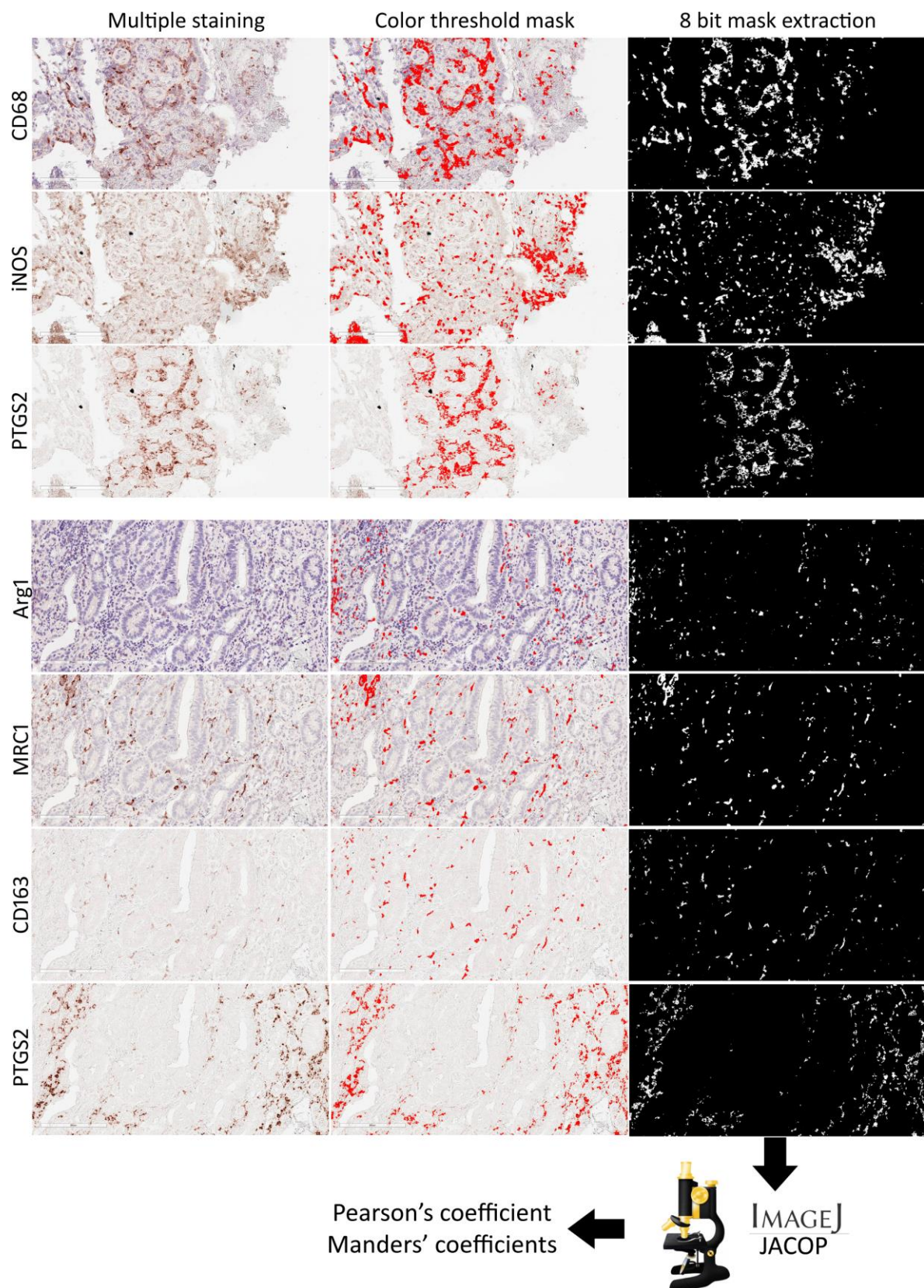

**Supplementary Figure S1.** Example of multiplex IHC and method for overlay computation.

Supplement: Supplementary file 1 [file cells-09-00683-s001.zip › Suppl files/Supplementary Figure S1.pdf]
